# Ca^2+^-modulated ROS-GC1 transduction system in testes and its presence in the spermatogenic cells

**DOI:** 10.3389/fnmol.2014.00034

**Published:** 2014-04-29

**Authors:** Anna Jankowska, Rameshwar K. Sharma, Teresa Duda

**Affiliations:** ^1^The Unit of Molecular Biology, Department of Cell Biology, Poznan University of Medical SciencesPoznan, Poland; ^2^The Unit of Regulatory and Molecular Biology, Research Divisions of Biochemistry and Molecular Biology Salus UniversityPA, USA

**Keywords:** testes, neurocalcin δ, membrane guanylate cyclase, ROS-GC1, calcium ions

## Abstract

ROS-GC1 belongs to the Ca^2+^-modulated sub-family of membrane guanylate cyclases. It primarily exists and is linked with signaling of the sensory neurons – sight, smell, taste, and pinealocytes. Exceptionally, it is also present and is Ca^2+^-modulated in t he non-neuronal cells, the sperm cells in the testes, where S100B protein serves as its Ca^2+^ sensor. The present report demonstrates the identification of an additional Ca^2+^ sensor of ROS-GC1 in the testes, neurocalcin δ. Through mouse molecular genetic models, it compares and quantifies the relative input of the S100B and neurocalcin δ in regulating the Ca^2+^ signaling of ROS-GC1 transduction machinery, and via immunochemistry it demonstrates the co-presence of neurocalcin δ and ROS-GC1 in the spermatogenic cells of the testes. The suggestion is that in more ways than one the Ca^2+^-modulated ROS-GC1 transduction system is linked with the testicular function. This non-neuronal transduction system may represent an illustration of the ROS-GC1 expanding role in the trans-signaling of the neural and non-neural systems.

## INTRODUCTION

Rod outer segment membrane guanylate cyclase, ROS-GC1 (known also as Ret-GC1 or GC-E), belongs to the family of membrane guanylate cyclases. Its discovery and molecular characterization was a land mark event in the field of phototransduction (reviewed in Pugh et al., 1997; Koch et al., 2010) as it identified the source of cyclic GMP that serves as a second messenger of the LIGHT signal. It also impacted the entire membrane guanylate cyclase field by dividing the guanylate cyclase family into two subfamilies, one comprising the hormone receptor cyclases, and the other, cyclases modulated by intracellular [Ca^2+^]_i_ signals (reviewed in: Sharma, 2010). ROS-GC1 belongs to the second subfamily together with another rod outer segment guanylate cyclase ROS-GC2 (Ret-GC2 or GC-F), and the olfactory neuroepithelial guanylate cyclase, ONE-GC (GC-D; reviewed in: Sharma, 2010).

The best-documented physiological function of ROS-GC1 and ROS-GC2 is in the recovery phase of phototransduction, to return the illuminated photoreceptors to the dark, resting state. Illumination of photoreceptors leads to activation of cyclic GMP phosphodiesterase, depletion of cyclic GMP, closure of the cyclic GMP gated (CNG) channels, lowering the free Ca^2+^ concentration, and hyperpolarization of the plasma membrane (reviewed in: Pugh et al., 1997; Koch et al., 2010). The ROS-GCs task is to restore the dark-level of cyclic GMP allowing opening of the CNG channels, increase of Ca^2+^ influx, and depolarization of plasma membrane. Ca^2+^ concentration, thus, determines the activities of ROS-GCs but in an indirect way. Guanylate cyclase activating proteins (GCAPs; GCAP1 and GCAP2) sense the post-illumination fall in Ca^2+^ and stimulate ROS-GCs to synthesize cyclic GMP at a faster rate and restore its dark level (reviewed in: Detwiler, 2000; Koch et al., 2010).

GCAPs, however, are not the only Ca^2+^ sensing modulators of ROS-GC activity. While increasing Ca^2+^ concentrations inhibit ROS-GCs activity through GCAPs, two other Ca^2+^ sensors, S100B and neurocalcin δ stimulate ROS-GC1 in a Ca^2+^-dependent fashion (Pozdnyakov et al., 1995; Margulis et al., 1996; Duda et al., 1996a; Kumar et al., 1999). The Ca^2+^-dependent S100B-mediated activation of ROS-GC1 operates in cones including their outer segments and pedicles (Duda et al., 2002; Wen et al., 2012). Its role in photo- and visual transductions remains to be established, but existing data indicate its involvement in transmission of the visual signal from cone ON-bipolar cells (Wen et al., 2012). Ca^2+^ signaling of ROS-GC1 activity mediated by neurocalcin δ is operative in retinal ganglion cells (Krishnan et al., 2004); and of ONE-GC in the olfactory neuroepithelium (Duda et al., 2001a, 2004).

Beyond the retina, ROS-GC1 is expressed in the pineal gland where in one subset of pinealocytes it co-localizes with GCAP1 and in another, with S100B (Venkataraman et al., 2000); in the mitral cells of the olfactory bulb where it co-localizes with GCAP1 (Duda et al., 2001b); and it co-immunoprecipitates with S100B in the gustatory epithelium (Duda and Sharma, 2004).

Neurocalcin δ (NCδ) belongs to a subfamily of neuronal calcium sensor (NCS) proteins called visinin like proteins (VSNLs). Similar to other, but not all NCS proteins, it is acylated at the N-terminus by myristic acid and undergoes a classical calcium-myristoyl switch (Ladant, 1995) e.g., it buries the myristoyl group in a hydrophobic pocket in a Ca^2+^ free form and exposes it in Ca^2+^-bound form, a phenomenon first observed for recoverin (Zozulya and Stryer, 1992). Myristoylation of neurocalcin δ allows its membrane association in response to changes in the intracellular Ca^2+^ concentration. However, once NCδ binds to the cellular membranes in a Ca^2+^-dependent fashion, part of it remains membrane associated even after removing Ca^2+^ by the addition of ethylene glycol tetraacetic acid (EGTA; Krishnan et al., 2004). Although the highest level of NCδ has been detected in neuronal tissues, its expression in the periphery is also observed. Functionally, NCδ has been linked to a wide variety of processes such as receptor endocytosis through interaction with α- and β-clathrin and β-adaptin (Ivings et al., 2002), trafficking and membrane delivery of glutamate receptors of the kainate type (Coussen and Mulle, 2006), and with microtubule assembly (Iino et al., 1995). The presence of NCδ has been found in the inner plexiform layer of the retina, e.g., in the amacrine and ganglion cells (Krishnan et al., 2004), olfactory sensory neurons (Duda et al., 2001b, 2004) and in type II cells of mouse circumvallate taste papillae (Rebello et al., 2011).

In the inner retinal layer NCδ acts as Ca^2+^-dependent modulator of membrane guanylate cyclase ROS-GC1 (Krishnan et al., 2004) and in the olfactory neuroepithelium, of ONE-GC (Duda et al., 2001a, 2004). The exact physiological significance of the ROS-GC1-NCδ signaling system in the retina is not known yet, it has, however, been proposed that the system may be involved in synaptic processes (Krishnan et al., 2004). In the olfactory neuroepithelium neurocalcin δ has been proposed as a Ca^2+^ sensor component of the two-step odorant uroguanylin signaling machinery (Duda and Sharma, 2009).

Outside the neuronal system, NCδ is expressed in the adrenal glomerulosa cells (Duda et al., 2012a,b). There it co-localizes with the member of receptor guanylate cyclase sub-family, atrial natriuretic factor receptor guanylate cyclase (ANF-RGC), and has been proposed to be involved in the inhibition of aldosterone synthesis (Duda et al., 2012a,b). Accordingly, a mouse model in which one copy of NCδ gene is deleted is inflicted with hyperaldosteronism and hypertension (Duda et al., 2012b).

Preliminary studies localize also ROS-GC1 outside the neuronal system, in male gonads, where it co-localizes with GCAP1 and S100B (Jankowska et al., 2007). It remains to be determined, whether, and how ROS-GC1 and its companion calcium binding proteins are involved in the physiology of the testis. Considering, however, that cyclic GMP and Ca^2+^ are genuine mediators of signal transduction in the testes in the physiology of fertilization (reviewed in: Garbers, 1989; Jankowska and Warchol, 2010) it is highly possible that ROS-GC1, stimulated or inhibited as needed, in a Ca^2+^-dependent fashion by Ca^2+^-binding proteins, is the provider of the physiologically necessary quantities of cyclic GMP. We now present new observations on co-presence of NCδ and ROS-GC1 in the spermatogenic cells and Ca^2+^-dependent modes of NCδ and S100B in modulating ROS-GC1 signaling the mammalian testes.

## MATERIALS AND METHODS

### TISSUES

The study group consists of testes from human (obtained from consenting organ donors, *n* = 5), bovine (purchased from local slaughter house, *n* = 3), and Wistar rat (obtained form the Vivarium of Poznan University of Medical Sciences, Poznan, Poland, *n* = 12). The study was approved by the IACUC and Ethics Review Boards of all the involved Institutions.

### GENETICALLY MODIFIED MICE

Care of the experimental animals conformed to the protocols approved by the IACUC at Salus University and was in strict compliance with the NIH guidelines. Construction of heterozygous NCδ-KO (NCδ^+/-^) mice is described in (Duda et al., 2012b). The S100B-KO mice are described in (Wen et al., 2012).

### ANTIBODIES

Rabbit ROS-GC1 and NCδ antibodies were produced, characterized and affinity purified as in (Venkataraman et al., 2003; Krishnan et al., 2004). Secondary antibodies, AP-conjugated goat anti-rabbit IgG, preimmune rabbit serum, NBT/BCIP, and Cy3-conjugated sheep anti-rabbit IgG were purchased from Sigma-Aldrich.

### REVERSE TRANSCRIPTION POLYMERASE CHAIN REACTION (RT-PCR)

Total RNA was isolated from seminiferous tubules and interstitial tissue using TriPure isolation reagent (Roche Diagnostics), according to the manufacturer’s protocols. The cDNA library was constructed using Advantage RT for PCR kit (BD-Bioscience) and used for the amplification of ROS-GC1 and neurocalcin δ fragments. Sequences of primers used for the amplification are listed in **Table 1**. The amplified fragments were purified on agarose gel and sequenced to confirm their identities.

**Table 1:** Primers used in PCR.

Species	Gene	PCR product	Primer sequence 5′	Primer sequence 3′	Amplified sequence
Human (*Homo sapiens)*	ROS-GC1 Kin	553 bp	5′GGGAATAAGGTATCTGCACC3′	5′CCGGATCAGATCCTCCA3′	nt: 2028–2581 NM_000180
	NCALD	526 bp	5′ACAGCAAGCTGCGCCCGGAGGTC3′	5′ACAATGGACGGGTCGCTATTGGC3′	nt:407-933 NM_001040624
Rat (*Rattus Norvegicus)*	ROS-GC1 Kin	566 bp	5′GAGATATCTCCACCATCGT3′	5′TAACTCCTCTGTGCGTTCT3′	nt: 2034-2600 L36029
	NCALD	526 bp	5′ACAGCAAGCTGCGCCCTGAAGTC 3′	5′ACAATGGAAGGGTCGCTTTTGGC3′	nt: 177-704 NM_001024371
Bovine (*Bos Taurus*)	ROS-GC1 Kin	548 bp	5′ATAAGGTATCTGCACCATCGAG3′	5′CCGGATCAGGTCCTCCAGGTTC5′	nt: 2026-2574 NM_174548
	NCALD	527 bp	5′ACAGCAAGCTGCGCCCGGAGGTCA3′	5′ACAATGGACGGGTCGCTCTTGGC3′	nt:25-551 NM_174398

### WESTERN BLOTTING

The procedure was carried out according to the previously published protocols (Venkataraman et al., 2000; Duda and Sharma, 2004; Jankowska et al., 2007). 150 mg of membrane fraction proteins were denatured in gel-loading buffer [62.5 mM Tris-HCl (pH 7.5), 2% SDS, 5% glycerol, 1 mM β-mercaptoethanol, and 0.005% bromophenol blue] in 95°C for 10 min. Samples were then subjected to SDS-PAGE in a buffer containing 0.025 mM Tris-HCl (pH 8.3), 0.192 M glycine, and 0.1% SDS. Afterwards the resolved proteins were transferred to nitrocellulose membranes. To avoid non-specific interactions membranes were incubated in Tris buffered saline containing 0.05% Tween 20 (TBS-T), 5% non-fat milk (blocking buffer) overnight at 4°C. ROS-GC1 and NCδ proteins were detected with specific rabbit polyclonal antibodies diluted 1:1500 and 1:1000, respectively. After 1 h incubation the blot was rinsed three times with TBS-T and incubated with anti-rabbit secondary antibodies conjugated with horseradish peroxidase (1:10 000).

To visualize the immunoreactive bands SuperSignal blaze chemiluminescent substrate (Pierce) was used according to the manufacturer’s protocol. Signal was detected by exposing the blot to Kodak X-ray film for 15 s. Then the X-ray film was scanned and processed using Photoshop 6.0 software.

### IMMUNOHISTOCHEMISTRY

Paraffin sections of the testes fixed in 4% paraformaldehyde were used for immunohistochemical detecting of ROS-GC1 and NCδ. To block the non-specific binding the sections were first washed with TBS (100 mM Tris-HCl, 0.9% NaCl) and incubated in blocking solution consisting of TBS buffer containing 0.05% Tween 20 (TBS-T) and 1% BSA for 1 h at room temperature. After washing with TBS-T the sections were incubated with the respective primary antibodies for 60 min at 37°C and washed four times (15 min each) in TBS-T. Anti ROS-GC1 antibodies were diluted 1:200 and anti neurocalcin δ, 1:100. AP-conjugated anti-rabbit IgG, diluted 1:200 and NBT/BCIP as the substrate were used for detection. Controls included detection reactions carried out under identical conditions except that the primary antibodies were replaced by preimmune serum.

### ISOLATION OF THE PARTICULATE FRACTION OF THE TESTES

Membrane fraction of the testes was isolated according to the protocol described previously (Marala and Sharma, 1988). The tissue was homogenized in a buffer containing 250 mM sucrose, 10 mM Tris-HCl (pH 7.4) and protease inhibitors. The homogenate was centrifuged at 400 *g* and then the supernatant at 10,000 *g* and finally, at 40,000 *g.* The resulting pellet represented the membrane fraction.

### GUANYLATE CYCLASE ACTIVITY ASSAY

The particulate fraction was assayed for guanylate cyclase activity as described previously (Paul et al., 1987; Jankowska et al., 2007, 2008). Briefly, membranes were preincubated on an ice-bath with or without NCδ in an assay system containing 10 mM theophylline, 15 mM phosphocreatine, 20 μg creatine kinase, and 50 mM Tris-HCl (pH 7.5), adjusted to the appropriate free Ca^2+^ concentration with precalibrated Ca^2^/EGTA solutions (Molecular Probes). The reaction was initiated by the addition of a substrate solution (4 mM MgCl_2_ and 1 mM GTP, final concentration) and continued by incubation at 37°C for 10 min. The reaction was terminated by the addition of 50 mM sodium acetate (pH 6.2) followed by heating in a boiling water-bath for 3 min. The amount of cyclic GMP formed was determined by radioimmunoassay (Nambi et al., 1982; Jankowska et al., 2007, 2008).

### STATISTICAL ANALYSES

The activity of guanylate cyclase was calculated as mean of at least six separate values ± SD.

## RESULTS

General consensus has been that ROS-GC1 is expressed exclusively in the sensory as well as in the second order neurons of the retina and in the neurons of the pineal gland and the olfactory bulb (Hayashi and Yamazaki, 1991; Goraczniak et al., 1994; Liu et al., 1994; Yang et al., 1995; Venkataraman et al., 2000; Duda et al., 2001a). However, earlier results of these investigators provided the first indication that ROS-GC1 is also expressed outside the neuronal system, in bovine testes and sperm (Jankowska et al., 2007, 2008). In the sperm, it is co-expressed with calcium sensor S100B and neuronal calcium sensor proteins GCAP1 and NCδ (Jankowska et al., 2008). These results were striking enough to warrant more detailed studies. Present investigation represents a step in that direction.

### MAMMALIAN TESTES EXPRESS ROS-GC1 AND NEUROCALCIN δ: ANALYSES AT THE mRNA LEVELS

The presence of ROS-GC1 and NCδ transcripts was analyzed in human, bovine, and rat testes. Using total RNA isolated from these tissues individual cDNA libraries were constructed and used for amplification of specific fragments of ROS-GC1 and NCδ cDNAs by polymerase chain reaction. For the amplification, distinct, species-specific primers for ROS-GC1 or NCδ were designed. These primers corresponded to the sequences located within so called “kinase-like domain” of ROS-GC1 cDNA and for NCδ they overlapped with the 5′- and 3′- ends of its coding sequence. The amplification yielded fragments of 553, 548, and 566 bp of the human, bovine, and rat ROS-GC1, respectively, and 526 bp fragment of neurocalcin δ (**Table 1**). Sequencing of all amplified products confirmed their identities with ROS-GC1 or NCδ cDNAs. The ROS-GC1 fragments represented indeed sequence coding for the kinase-like domain, and the NCδ fragment had sequence identical with the predicted part of NCδ coding region. Amplification of uninterrupted coding sequences for ROS-GC1 and NCδ documented that the RNA used was not contaminated with genomic DNA. Thus both, ROS-GC1 and NCδ transcripts are present in human, bovine, and rat testes.

### MAMMALIAN TESTES EXPRESS ROS-GC1 AND NEUROCALCIN δ: ANALYSES AT THE PROTEIN LEVELS

At the protein level, the expression of ROS-GC1 and NCδ in mammalian testes was tested in two ways. First, a rudimentary assessment of the expression of both types of proteins was obtained by Western blotting; this was followed by immunocytochemical localization of ROS-GC1 and NCδ in human and rat testes. In both types of analyses appropriate affinity purified antibodies, anti-ROS-GC1, or anti-NCδ were used. Immunostaining of bovine testis was performed in parallel as positive control.

**FIGURE 1:**
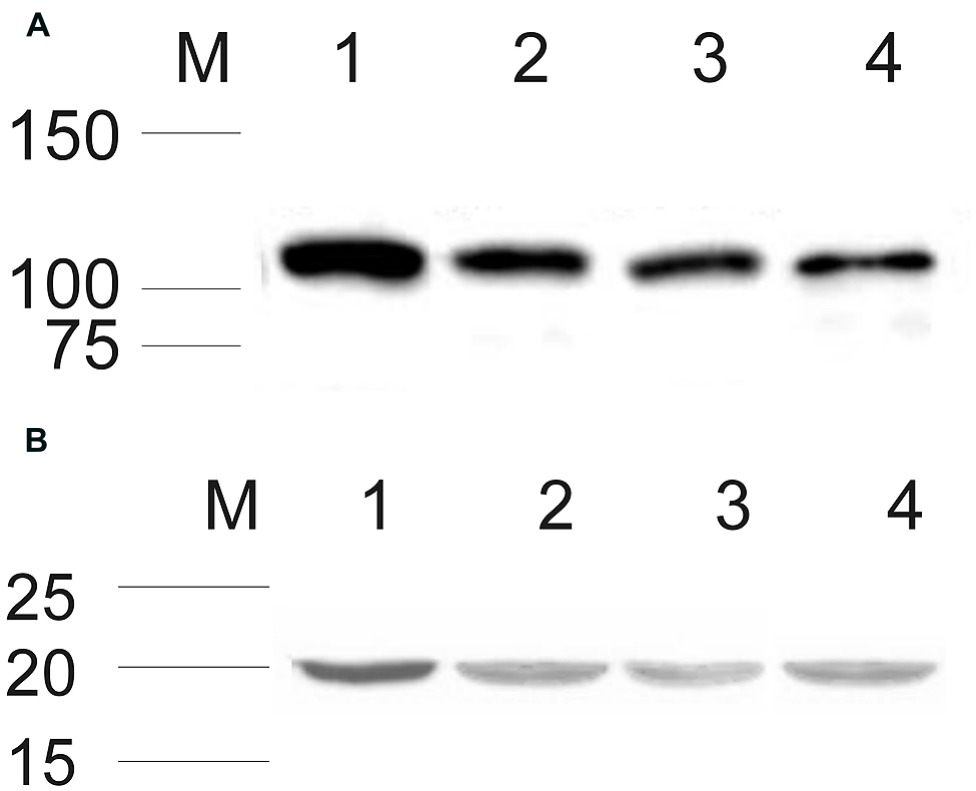
**Expression of ROS-GC1 and NCδ in the testes analyzed by Western blot.** Membrane proteins from human, rat, and bovine testes were isolated and subjected to Western blot analyses with specific antibodies against ROS-GC1 **(A)**, and NCδ **(B)** as described in “Materials and Methods.” Both types of analyzed proteins expressed in human, rat, and bovine testis are shown in lanes 2, 3, and 4, respectively. Western blotting of bovine retinal membranes was performed as a positive control and is shown in lane 1 in **(A)** and **(B)**. The positions of the molecular size markers are given alongside.

After establishing that ROS-GC1 and NCδ transcripts are present in mammalian testes, their protein identity was scrutinized by Western blotting. The mobility of the ROS-GC1 or NCδ immunoreactive band, ~116 kDa and ~20 kDa, respectively, (**Figures 1A,B**) was identical to that observed previously for ROS-GC1 expressed in photoreceptor outer segments (Duda et al., 1996b) and NCδ expressed in the inner retina (Krishnan et al., 2004). These results clearly confirm that both proteins, ROS-GC1 and NCδ are expressed in human, rat, and bovine testes.

**FIGURE 2:**
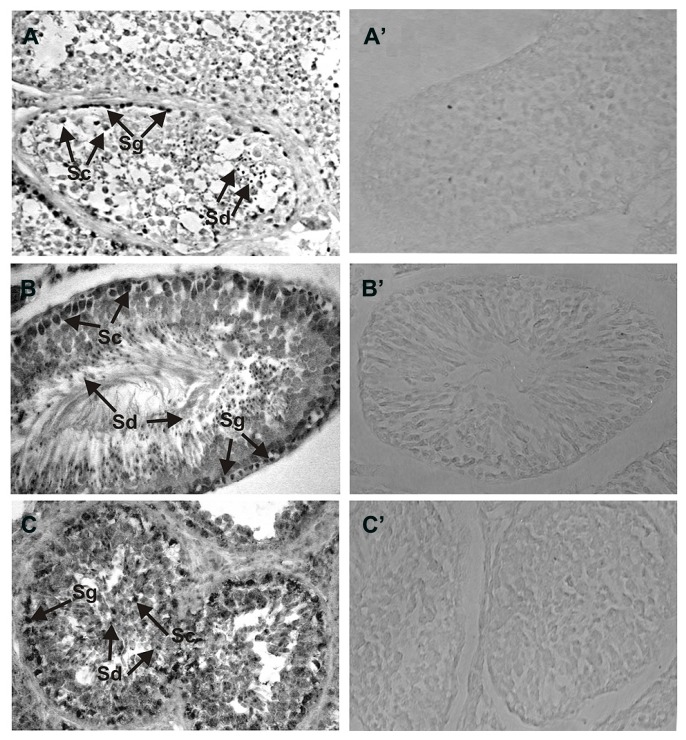
**Immunolocalization of ROS-GC1 in the testes.** Immunohistochemistry was performed using specific antibodies against ROS-GC1 on paraffin sections of human **(A)**, rat **(B)**, and bovine **(C)** testes. The ROS-GC1 was localized in spermatogenic cells. In the testes positive staining was observed in spermatogonias (Sg), spermatocytes (Sc) and spermatids (Sd), as indicated by the arrows. Control staining was performed according to the procedure for ROS-GC1 detection, except that preimmune serum was used instead of the primary antibody **(A’–C’)**. Original magnification 400X.

Detailed analyses of the localization of ROS-GC1 and NCδ in the mammalian testes were performed by immunocytochemistry. In human and rat testes ROS-GC1 immunostaining was randomly distributed in the seminiferous tubules (**Figures 2A,B**). The pattern of this staining was identical to that observed for bovine testes (**Figure 2C**). Visual analysis allowed localizing the staining to spermatogenic cells, especially to primary spermatocytes and spermatids. The majority of primary spermatocytes (**Figure 2**; indicated as Sc), spermatids (**Figure 2**; indicated as Sd) and single spermatogonias (**Figure 2**; indicated as Sg) were immunostained in all species tested, human, rat, and bovine. To verify specificity of the ROS-GC1 immunostaining a control reaction was performed in which ROS-GC1 antibody was omitted and substituted with the preimmune serum. Under these conditions no labeling was observed (**Figures 2A’–C’**).

**FIGURE 3:**
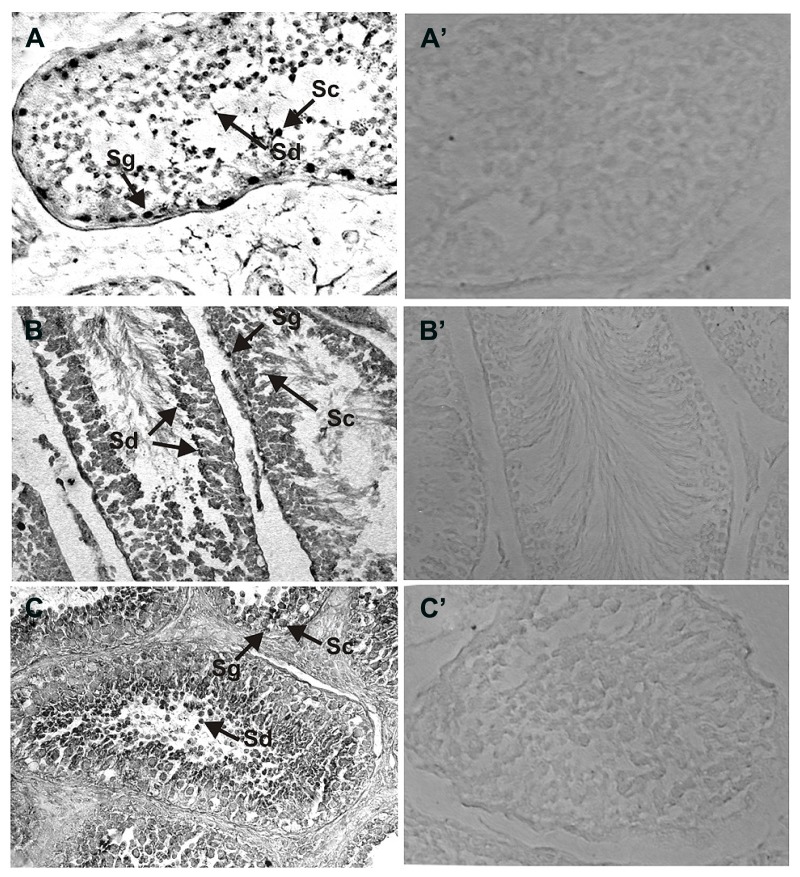
**Immunolocalization of NCδ in the testes.** Paraffin sections of the testes were incubated with primary antibodies against NCδ. NCδ immunoreactivity was observed in all spermatogenic cells of human **(A)**, rat **(B)**, and bovine **(C)** testes. Particularly strong signal was detected in spermatocytes and spermatids localized close to lumen of the seminiferous tubule (indicated as Sc and Sd, respectively). Control staining was performed according to the procedure for NCδ detection, except that preimmune serum was used instead of the primary antibody **(A’–C’)**. Original magnification 400X.

In a similar manner the expression of NCδ in the human and rat testes was analyzed (**Figures 3A,B**) and compared with that in bovine testis (**Figure 3C**). NCδ immunoreactivity was observed in all types of germinal cells: spermatogonias (**Figure 3**: Sg), spermatocytes (**Figure 3**: Sc) and spermatids (**Figure 3**: Sd). Extremely strong staining was observed in spermatids localized close to seminiferous tubule lumen. No labeling was observed when the sections were incubated with preimmune rabbit serum instead of the NCδ antibody, attesting to the specificity of the staining (**Figures 3A’–C’**).

The results presented clearly demonstrate that both ROS-GC1 and NCδ are expressed in the mammalian testes and in the germinal cells. Importantly, the patterns of ROS-GC1 and NCδ immunoreactivities are identical in the species analyzed (compare **Figures 2** and **3**) strongly indicating that they co-localize with each other. Notably, the intensities of the immunostaining for ROS-GC1 or NCδ increase with the maturation of the spermatogenic cells.

Since the increase in the staining is observed across the cells in different stages of spermatogenesis process – the highest accumulation of analyzed proteins was observed in spermatocytes and spermatids – it indicates that ROS-GC1 and NCδ expression correlates with the stage of the seminiferous cycle.

### NEUROCALCIN δ STIMULATES ROS-GC1 CATALYTIC ACTIVITY EXPRESSED IN THE TESTES: EVIDENCE FROM RECONSTITUTION EXPERIMENTS

**FIGURE 4:**
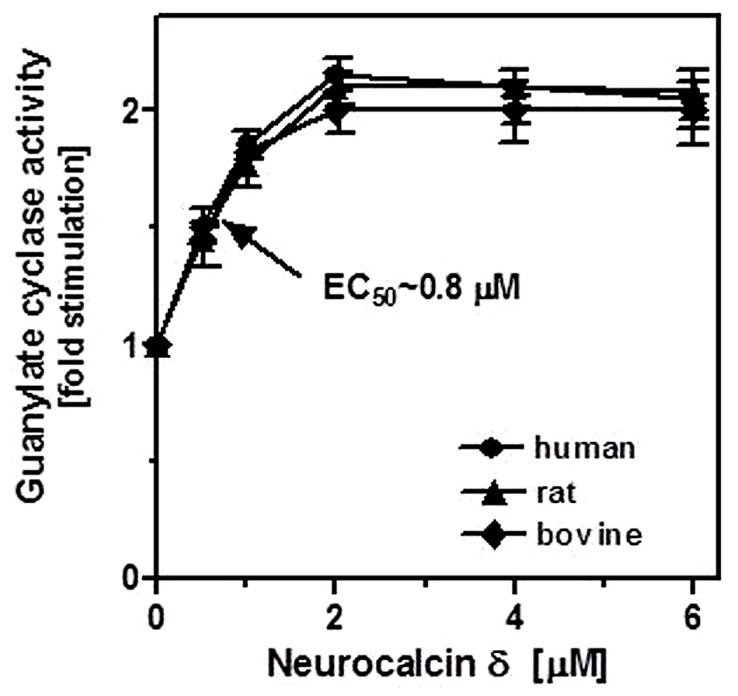
**Effect of NCδ on the guanylate cyclase activity in the membranes of testes.** Membranes of human, rat, and bovine testes were isolated as described in “Materials and Methods”. They were assayed for guanylate cyclase activity in the presence of indicated concentrations of NCδ and 100 μM Ca^2+^. Each experiment was done in triplicate, and repeated two times. The results shown (mean ± SD) are from these experiments.

To demonstrate that neurocalcin δ stimulates ROS-GC1 expressed in the testes, their membranes were analyzed for guanylate cyclase activity in the presence of NCδ. Membranes of human, bovine, and rat testes were isolated and incubated with increasing concentrations of NCδ and constant 100 μM Ca^2+^. The amount of cyclic GMP formed was determined as a measure of guanylate cyclase activity. NCδ stimulated the cyclase activity in a dose-dependent fashion (**Figure 4**). Half-maximal stimulation (EC_50_) was observed at ~0.8 μM NCδ in all three species tested. The maximal stimulation of ~2.2-fold above the basal value was at 2 μM NCδ. Importantly, the stimulatory profiles of guanylate cyclase in the analyzed membranes were very similar to those observed for the recombinant ROS-GC1 (Kumar et al., 1999) and ROS-GC1 native to the retinal photoreceptor outer segments and inner plexiform layer (Krishnan et al., 2004). These results show that NCδ indeed stimulates ROS-GC1 activity in the testes.

### ROS-GC1 AND NCδ ARE FUNCTIONALLY LINKED: EVIDENCE FROM GENETICALLY MODIFIED MICE

**FIGURE 5:**
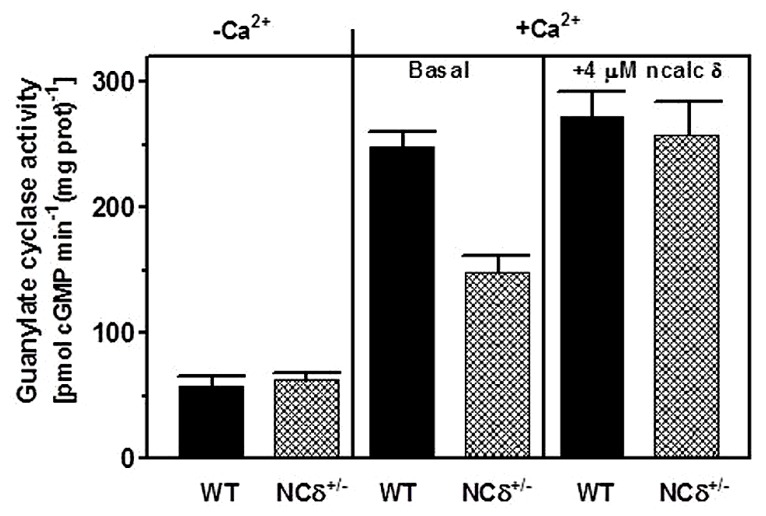
**NCδ modulates ROS-GC1 activity in mouse testes.** Testes were removed from the wild type (WT) and NCδ^+/-^ mice and their particulate fractions were isolated in the presence of 100 μM Ca^2+^. These were assayed for guanylate cyclase activity in the presence of 1 mM EGTA or 10 μM Ca^2+^ (panel “-Ca^2+^”- and “+Ca^2+^”-basal). 4 μM myristoylated NCδ was added to the membranes and the guanylate cyclase activity was assessed in the presence of 10 μM Ca^2+^. The experiment was done in triplicate and repeated with separate membrane preparations. The results are mean ± SD of these experiments.

Because the experiments described above were conducted on the native membranes of the testes but with exogenously supplied NCδ, they prove the ability of ROS-GC1 to respond to NCδ but they do not prove that these two together form a functional unit. To determine this, the mouse model with deletion of one copy of the NCδ gene, NCδ^+/-^, was used [mice with deletion of both copies of NCδ are not born (Duda et al., 2012b)]. It was reasoned that if NCδ is indeed the Ca^2+^-sensor modulator of ROS-GC1 in the testes of these mice the NCδ-modulated Ca^2+^ signaling pathway should be half as active as in those of the wild type mice [wt (NCδ^+/+^)]. To test this prediction, the particulate fractions of the testes from wild type and NCδ^+/-^ mice were isolated in the presence of 100 μM Ca^2+^ and tested for guanylate cyclase activity in the presence and absence of Ca^2+^. The reason for isolating the membranes in the presence of Ca^2+^ was that NCδ exhibits the property of “calcium-myristoyl switch” which means that in the presence of Ca^2+^ it is membrane bound and therefore would be present in the membrane fraction (Ladant, 1995; Krishnan et al., 2004). The guanylate cyclase activity assayed in the absence of Ca^2+^ (1 mM EGTA was present in the assay mixture) was 58 ± 7 pmol cyclic GMP min^-1^ (mg prot)^-1^ for the wt and 63 ± 8 pmol cyclic GMP min^-1^ (mg prot)^-1^ for the NCδ^+/-^ mice (**Figure 5**: panel “Ca^2+^”). However, the cyclase activity determined in the presence of Ca^2+^ was strongly dependent on the mice genotype. With 1 μM Ca^2+^ present in the assay mixture the activity was 248 ± 17 pmol cyclic GMP min^-1^ (mg prot)^-1^ for the wild type mice and 148 ± 16 pmol cyclic GMP min^-1^ (mg prot)^-1^ for the NCδ^+/-^ mice (**Figure 5**: panel “+Ca^2+^ – basal”). These results, as predicted, demonstrate that the Ca^2+^-dependent NCδ-modulated ROS-GC1 signaling pathway in the mice with one copy of NCδ gene deleted (NCδ^+/-^) is about half as active as in the wild type mice. To further validate that the lowering of the Ca^2+^-dependent cyclase activity in the membranes of NCδ^+/-^ mice is the exclusive consequence of lower NCδ expression, 4 μM exogenous NCδ was added to the wild type and NCδ^+/-^ membranes and the cyclase activity was assessed in the presence of 1 μM Ca^2+^. The cyclase activity in the wild type membranes increased only minimally, from 248 to 273 ± 25 pmol cyclic GMP min^-1^ (mg prot)^-1^but in the NCδ^+/-^ membranes, from 148 to 258 ± 28 (**Figure 5**: panel “+Ca^2+^ + 4 μM NCδ”). Thus, the activity achieved was practically the same for both types of membranes. These results demonstrate that addition of exogenous NCδ to the NCδ^+/-^ membranes restores the guanylate cyclase catalytic activity and brings it to the level of activity in the wild type membranes. The slight activity increase in the wild type membranes can be explained by a partial loss of the native NCδ during the membrane preparation procedure. Together these results demonstrate that functional ROS-GC1-NCδ-Ca^2+^ transduction system is operative and is the functional transduction element in the testes.

### WHICH CA^2+^ SENSOR, NCδ OR S100B, IS THE MORE POTENT REGULATOR OF ROS-GC1 ACTIVITY?

Our previous investigation has shown that S100B is expressed in the testes (Jankowska et al., 2007) and the present results demonstrate that NCδ is also expressed there. Thus, two Ca^2+^-dependent modulators of ROS-GC1 activity are present in the testes. Taking advantage of the availability of two types of genetically modified mice, NCδ^+/-^ and S100B^-/-^ we attempted comparing the relative inputs of NCδ and S100B into regulation of ROS-GC1 transduction machinery.

**FIGURE 6:**
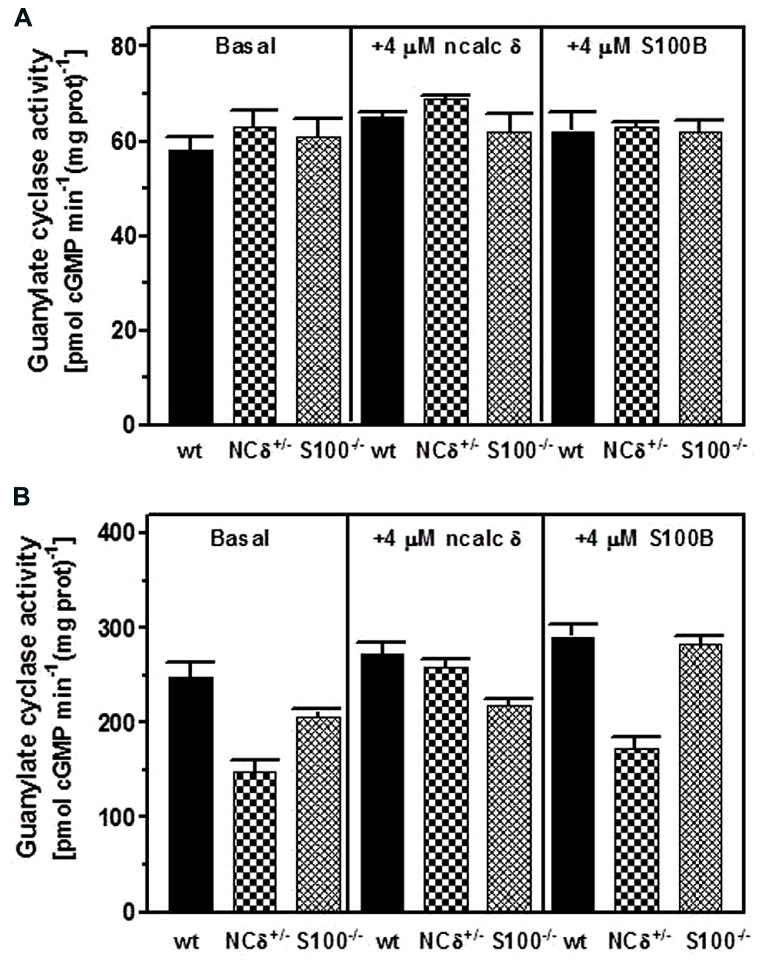
**Comparison of NCδ and S100B stimulatory effects on ROS-GC1 activity in mouse testes.** Testes were removed from the wild type (WT), NCδ^+/-^, and S100^-/-^ mice and their particulate fractions were isolated in the presence of 100 μM Ca^2+^. **(A)** The membranes were assayed for guanylate cyclase activity in the presence of 1 mM EGTA in the absence (panel “basal”) or presence of 4 μM myristoylated NCδ (panel (“+ 4 μM ncalc δ”) or 4 μM S100B (panel “+S100B”). **(B)** The membranes were assayed for guanylate cyclase activity in the presence of 10 μM Ca^2+^(“basal”) or with the addition of 4 μM NCδ (panel “+ 4 μM ncalc δ”) or 4 μM S100B (panel “+ 4 μM S100B”). The experiment was done in triplicate and repeated with separate membrane preparations. The results are mean ± SD of these experiments.

Testes were removed from the wild type, NCδ^+/-^ and S100B^-/-^ mice, their membrane fractions were isolated in the presence of 100 μM Ca^2+^ and assessed for guanylate cyclase activity. The results of this experiment are shown in **Figure 6**. In the absence of Ca^2+^ all three types of membranes exhibited comparable activity of ~60 pmol cyclic GMP min^-1^ (mg prot)^-1^ (**Figure 6A**: panel “basal”) and, as expected, the activity was not affected by the addition of NCδ or S100B (**Figure 6A**: panels “+ncalc δ” and “+S100B”). However, when the cyclase activity was assayed in the presence of Ca^2+^ the values differed between the genotypes: 250 pmol cyclic GMP min^-1^ (mg prot)^-1^ for the wild type, 150 pmol cyclic GMP min^-1^ (mg prot)^-1^ for the NCδ^+/-^ and 200 pmol cyclic GMP min^-1^ (mg prot)^-1^ for the S100B^-/-^ (**Figure 6B**: panel “basal”). These values describe the ROS-GC1 activity stimulated together by NCδ and S100B in the case of the wild type membranes; stimulated by half of wild type amount of NCδ and S100B in the case of NCδ^+/-^ membranes; and stimulated by NCδ only in the case of S100B^-/-^ membranes. Thus, the difference between ROS-GC1 activity in testes of the wild type mouse and the activity in the genetically modified mice (100 and 50 pmol pmol cyclic GMP min^-1^ (mg prot)^-1^ for the NCδ^+/-^ and S100B^-/-^, respectively) describes the Ca^2+^-dependent stimulatory effects lost due to the absence of half of the NCδ present in the wild type testes or to the total absence of S100B. Higher loss of activity resulting from deletion of one copy of NCδ gene than resulting from deletion of two copies of S100B gene indicates that NCδ is more potent than S100B in modulating the Ca^2+^-dependent ROS-GC1 activity. One could argue, however, that this comparison might not be totally reflective of the *in vivo* contributions of NCδ and/or S100B, because there is a possibility that some of these proteins were lost during the membrane preparation process. To settle this possibility the ROS-GC1 activity was measured in the presence of individually added NCδ or S100B.

Addition of 4 μM NCδ (**Figure 6B**: panel “+ncalc δ”) resulted in a small increase of the cyclase activity exhibited by the membranes isolated from the wild type testes (from ~250 to ~270 pmol cyclic GMP min^-1^ (mg prot)^-1^ and isolated from the S100B^-/-^ testes [from ~200 to ~220 pmol cyclic GMP min^-1^ (mg prot)^-1^]. This non-significant to minimal increase of ROS-GC1 catalytic activity in these membranes indicates that if any NCδ was lost during the membrane preparation, it was only the insignificant amount. For the NCδ^+/-^ membranes the increase was significant, from ~150 to ~260 pmol cyclic GMP min^-1^ (mg prot)^-1^. Importantly, addition of 4 μM NCδ brought the ROS-GC1 activity in these membranes very close to the ROS-GC1 activity in the wild type membranes. Because only residual amount of NCδ was lost during membrane preparation, as concluded from the wild type and S100B^-/-^ membranes, practically all the increase of activity in the NCδ^+/-^ membranes accounts for the activity lost due to the expression of only half of the NCδ present in the membranes from wild type testes.

When the ROS-GC1 activity was assayed with 4 μM S100B added to the membranes (**Figure 6B**: panel “+S100B”) it increased by ~40 pmol cyclic GMP min^-1^ (mg prot)^-1^ in the wild type and by 25 pmol cyclic GMP min^-1^ (mg prot)^-1^in the NCδ^+/-^ membranes indicating that some S100B was lost in membranes preparation. The increase was ~80 pmol cyclic GMP min^-1^ (mg prot)^-1^for the S100B^-/-^ membranes [from 205 to 282 pmol cyclic GMP min^-1^ (mg prot)^-1^] and this increase of activity in the S100B^-/-^ membranes accounts for the activity lost due to the loss of S100B expression in the testes. Comparing the activity loss because of the absence of half of NCδ with the activity loss due to the total absence of S100B expression shows that neurocalcin δ is more significant contributor to ROS-GC1 Ca^2+^-dependent activity than S100B. It could be estimated that NCδ contributes about 75% to ROS-GC1 Ca^2+^-dependent activity and S100B, about 25%.

## DISCUSSION

The core finding of this study is that it establishes the presence and molecular nature of a Ca^2+^- modulated ROS-GC transduction system in all types of the germinal cells of the testes: spermatogonias, spermatocytes, and spermatids. Because abundance of this transduction system is present in the spermatids residing close to the seminiferous tubule lumen, it hints that the system is linked with the processes of spermatogenesis.

This Ca^2+^-dependent cyclic GMP generating transduction system is composed of a transducer component ROS-GC1 and two Ca^2+^ sensor components, NCδ and S100B. NCδ is the major signal contributor responsible for about 75% of the guanylate cyclase stimulated catalytic activity, and S100B the relatively minor contributor, about 25% of signaling activity.

Cyclic GMP appears to be a critical second messenger in the physiology of the testes. In particular, it has been shown to influence motility in spermatozoa, development of testicular germ cells, relaxation of peritubular lamina propia cells, testosterone synthesis in Leydig cells, and dilatation of testicular blood vessels (Singh et al., 1988; Armstrong et al., 1994; Middendorff et al., 1997, 2000). Our immunostaining results demonstrate that both ROS-GC1 and NCδ immunoreactivity is present in all germ cells but its intensity is lowest in spermatogonias and highest in spermatids, hence, it increases with maturation, indicating that development of these cells may be directed by the activity of ROS-GC1.

The present study, of basic biochemical nature, brings to the fore several issues for future research. (1) What is the exact composition of the transduction system: does it represent a single pathway, composed of one ROS-GC1 and two Ca^2+^ sensors, NCδ, and S100B operating simultaneously? Or, two independent, but complementary pathways, each composed of one ROS-GC1 and one Ca^2+^ sensor, NCδ, or S100B? (2) What is the precise morphological residence of any of these transduction systems in the testicular cells? (3) What is the exact stoichiometry of these components in the given cell types? (4) What are the mechanistic details of the operational modes of this/these ROS-GC transduction pathways? (5) What is the physiological significance of these pathways to the testicular function, endocrine, fertility, including spermatogenesis; (6) what is the interplay between the Ca^2+^-modulated ROS-GC1 signaling pathway and ANF-dependent ANF-RGC pathway, another cyclic GMP generating machinery functioning in the mammalian testes (Marala and Sharma, 1988; Pandey and Singh, 1990; Pandey et al., 1999; Mourdjeva et al., 2001) and finally, (7) how this information can be translated to the clinical level to search for the pathway/s-linked diseases and finding their cures?

**FIGURE 7:**
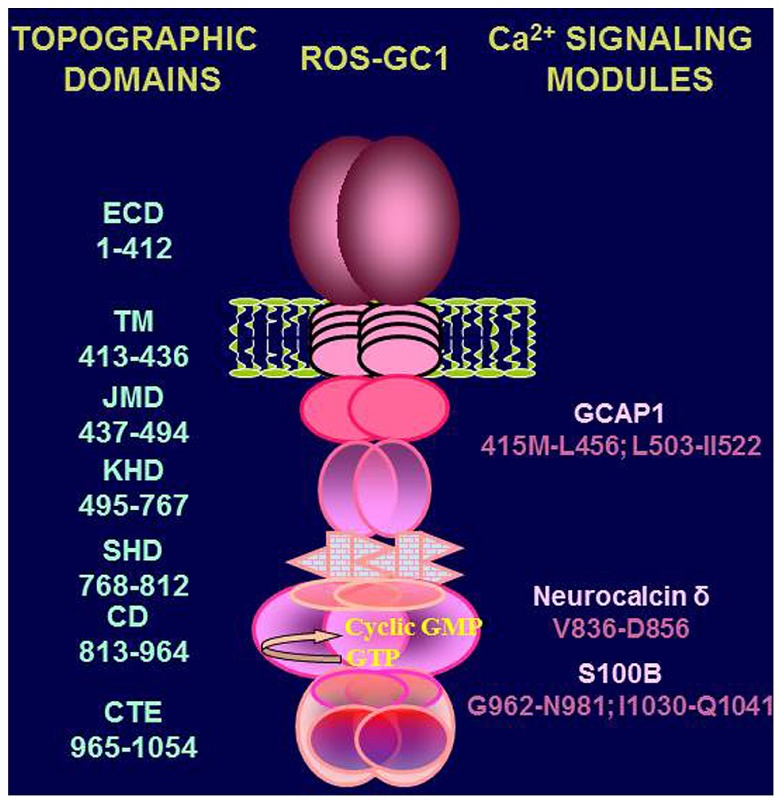
**Topography of ROS-GC1.** A schematic diagram of ROS-GC1, with its multiple domains is provided. The extracellular (ECD), the transmembrane (TM), the juxtamembrane (JMD), the kinase homology (KHD), the signalling helix (SHD), the catalytic (CD), and the C-terminal extension (CTE) domains have been identified on the left-hand side; the GCAP1, NCδ, and S100B binding domains are indicated to the right.

Handicapped with these informational gaps, it is difficult to construct a proper Ca^2+^-modulated signal transduction model for any of the specific physiological function/s of the testes. However, a preliminary signal transduction scheme is conceptualized where [Ca^2+^]_i_ modulation of ROS-GC1 activity can occur through three modes. MODE 1 involves interaction of ROS-GC1 with its Ca^2+^ sensors, NCδ and S100B. The ROS-GC is stimulated in [Ca^2+^]_i_ – dependent manner and generates cyclic GMP. MODE 2 involves interaction of the ROS-GC1 with GCAP1 which is also present in the testes (Jankowska et al., 2007). It is recalled that GCAP1 signaling is opposite to that of NCδ or S100B; it stimulates ROS-GC1 catalytic activity in the absence of Ca^2+^ and progressively inhibits it with increasing Ca^2+^ concentration (Duda et al., 1996b). Thus, through GCAP1 [Ca^2+^]_i_ keeps the ROS-GC activity in the suppressed state. MODE 3, it is the most interesting scenario. ROS-GC1 is present together with the Ca^2+^-dependent guanylate cyclase activators (CD-GCAPs)–NCδ, S100B – and with GCAP1. The ROS-GC activity oscillates between the GCAP1-dependent state and CD-GCAPs-dependent state. In these states the cyclase is a bimodal Ca^2+^ transduction switch. And it functions according to the principles established for the synapse region between the photoreceptor and ON-bipolar cells (Duda et al., 2002; Venkataraman et al., 2003). This mode of ROS-GC1 activity regulation is possible because each of the three modulators targets its individual domain in ROS-GC1. These domains have been mapped and are schematically shown in **Figure 7**. Neurocalcin δ targets the ROS-GC1 region V^836^–D^856^ (Venkataraman et al., 2008); S100B, G^962^–N^981^and I^1030^–Q^1041^ (Duda et al., 2002); GCAP1, M^445^–L^456^ and L^503^–1^522^(Lange et al., 1999).

### MODE 3 – MODEL

*Basal state – low Ca^2+^*: Ca^2+^-free GCAP1 stimulates ROS-GC1 catalytic activity. Cyclic GMP formed in response to this stimulation opens some of the CNG channels present in the sperm membranes (Wiesner et al., 1998; Revelli et al., 2002). At least some of the Ca^2+^ ions entering the sperm through the open CNG channels are bound by neurocalcin δ and/or S100B. It is important to note that the half-maximal activation of ROS-GC1 by GCAP1 occurs at 707 nM Ca^2+^ (Hwang et al., 2003), the Ca^2+^ concentration at which neurocalcin δ and S100B are able to activate ROS-GC1 with approximately half-maximal effectiveness. With increasing Ca^2+^ concentrations GCAP1 inhibits ROS-GC1 activity while neurocalcin δ and S100B stimulate it. *Active state* – Ca^2+^-bound NCδ and/or S100B further stimulate ROS-GC1 and cyclic GMP is formed in higher quantities leading to massive opening of CNG channels and influx of Ca^2+^ (Shapiro et al., 1990; Revelli et al., 2002). In this phase, the ROS-GC1 activity is resultant of two opposing effects, inhibitory through GCAP1 and stimulatory, through NCδ and S100B. *Signal termination*: when cyclic GMP and [Ca^2+^]_i_ reach the optimal level necessary for sperm membrane hyperpolarization and physiological function (e.g., capacitation and/or acrosome reaction; Galinado et al., 2000) combined effects of protein phosphorylation and phosphodiesterase activity (Su and Vacquier, 2006) lead to lowering of cyclic GMP levels, closure of the CNG channels and cessation of the Ca^2+^ influx.

## CONCLUSION

Co-expression of NCδ, S100B, and GCAP1 with ROS-GC1 in mammalian spermatogenic cells confers bimodal Ca^2+^ sensitivity to cyclic GMP synthesis. The stimulatory arm of the switch operated by NCδ and S100B is essential for timely opening of the CNG channels by cyclic GMP produced and vast influx of Ca^2+^ necessary for the acrosome reaction. In this stimulatory arm NCδ appears to be a significantly more important player than S100B. The inhibitory arm operated by GCAP1 stops the cyclic GMP synthesis and therefore Ca^2+^ influx. Our findings demonstrate that ROS-GC1 transduction system and its bimodal Ca^2+^ modulation is not unique to neurosensory and neurosensory-linked systems and has a more widespread role in general Ca^2+^-dependent signaling and may be a part of the fertilization machinery.

## Conflict of Interest Statement

The authors declare that the research was conducted in the absence of any commercial or financial relationships that could be construed as a potential conflict of interest.
